# Effective reduction of post‐inflammatory hyperpigmentation with the tyrosinase inhibitor isobutylamido‐thiazolyl‐resorcinol (Thiamidol)

**DOI:** 10.1111/ics.12694

**Published:** 2021-05-05

**Authors:** Dennis Roggenkamp, Ncoza Dlova, Tobias Mann, Jan Batzer, Julia Riedel, Martina Kausch, Ivica Zoric, Ludger Kolbe

**Affiliations:** ^1^ International Medical Management Eucerin Beiersdorf AG Hamburg Germany; ^2^ Nelson R Mandela School of Medicine Durban South Africa; ^3^ Research & Development, Beiersdorf AG Hamburg Germany

**Keywords:** emulsions, isobutylamido‐thiazolyl‐resorcinol, post‐inflammatory hyperpigmentation, skin physiology, spectroscopy, Thiamidol

## Abstract

**Objective:**

Post‐inflammatory hyperpigmentation (PIH) is a major cosmetic concern especially in individuals with darker skin complexion. Unfortunately, treatment with anti‐inflammatory ingredients alone does not prevent the development of hyperpigmented spots. Recently, isobutylamido‐thiazolyl‐resorcinol (Thiamidol) was described as a very potent inhibitor of human tyrosinase. The objective of this research was to investigate the potential of this compound to prevent PIH induced by epidermal wounding (suction blister) and related to acne.

**Methods:**

Suction blister‐induced PIH was treated with a formulation containing Thiamidol or a vehicle for 3 months, and the changes in hyperpigmentation were monitored by spectroscopic measurements. The effect of skin care formulations containing Thiamidol on acne‐related PIH was investigated in two studies, a vehicle‐controlled, double‐blinded, randomized clinical study and a clinical observational study. Both studies had a duration of 3 months and included assessments such as clinical photography, clinical grading and melanin index measurements.

**Results:**

Already after 2 weeks of treatment, suction blister sites treated with Thiamidol were significantly lighter than control sites and improved throughout the treatment period. Subjects´ self‐grading demonstrated that Thiamidol significantly improved the visibility of acne‐induced hyperpigmentation compared to the vehicle treatment. A skin care regimen with Thiamidol significantly improved acne‐related PIH over 12 weeks shown by Mexameter measurements, expert grading, self‐grading and clinical photography.

**Conclusion:**

Thiamidol represents a safe and effective ingredient for cosmetic products against post‐inflammatory hyperpigmentation.

## Graphical abstract

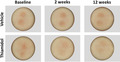

## Résumé

**Objectif:**

L’hyperpigmentation post‐inflammatoire (Post‐Inflammatory Hyperpigmentation, PIH) est une préoccupation d’ordre esthétique majeure, en particulier chez les personnes dont le teint est plus foncé. Malheureusement, un traitement par des ingrédients anti‐inflammatoires seuls n’empêche pas le développement de taches hyperpigmentées. Récemment, l’isobutylamido‐thiazolyl‐résorcinol (Thiamidol) a été décrit comme un inhibiteur très puissant de la tyrosinase humaine. L’objectif de cette investigation était d’étudier le potentiel de ce composé pour prévenir la PIH induite par une plaie épidermique (bulle de succion) et liée à l’acné.

**Méthodes:**

La PIH induite par la bulle de succion a été traitée avec une formulation contenant du Thiamidol ou un véhicule pendant 3 mois et les changements dans l’hyperpigmentation ont été surveillés par le biais de mesures spectroscopiques. L’effet des formulations pour soins de la peau contenant du Thiamidol visant à traiter la PIH liée à l’acné a été étudié au cours de deux études : une étude clinique randomisée, en double aveugle, contrôlée par véhicule et une étude clinique observationnelle. Les deux études avaient une durée de 3 mois et comportaient des évaluations telles que la photographie médicale, l’évaluation par les médecins et les mesures de l’indice de mélanine.

**Résultats:**

Après 2 semaines de traitement seulement, les sites de bulles de succion traités par du Thiamidol étaient significativement plus clairs que les sites‐témoins et avaient montré une amélioration tout au long de la période de traitement. L’auto‐évaluation des sujets a démontré que le Thiamidol améliorait significativement la visibilité de l’hyperpigmentation induite par l’acné par rapport au traitement par véhicule. Un programme de soins de la peau contenant du Thiamidol a significativement amélioré la PIH liée à l’acné sur 12 semaines, comme l’ont démontré les mesures de Mexameter, l’évaluation par des experts, l’auto‐évaluation des sujets et la photographie médicale.

**Conclusion:**

Le Thiamidol représente un ingrédient sûr et efficace pour les produits cosmétiques contre l’hyperpigmentation post‐inflammatoire.

## INTRODUCTION

The treatment of post‐inflammatory hyperpigmentation (PIH) is a major cosmetic challenge. Clinically, PIH skin is characterized by the appearance of irregular pigmentation extending from scattered, localized macules to large patches [1]. PIH is the consequence of cutaneous inflammatory insult. The exact mechanism leading to PIH is, however, still not completely understood [2]. Following skin inflammation, it is likely that either an overproduction of melanin and/or an irregular distribution of melanin pigment takes place [3, 4]. The inflammatory process can be triggered by different endogenous (e.g. acne vulgaris or atopic dermatitis) or exogenous (e.g. insect bites, laser or peeling procedures, slight injuries, burns, shaving irritations) factors [1, 4].

PIH affects all skin types but is more common in Fitzpatrick skin types III to VI and represents one of the most frequent diagnoses regarding pigmentation disorders in individuals from African, Asian and south American decent [1, 5]. Darker‐skinned individuals already exhibit a higher basal epidermal melanin concentration and an increased reactivity of melanocytes. In these subjects, hypermelanosis was shown to be more severe, more obvious and longer lasting [1, 3, 4, 6, 7]. Since PIH often develops in sun‐exposed areas of the body, especially the face, the emotional and psychological effects of PIH can have a strong negative impact on a patient’s quality of life [3].

For PIH treatment, topical agents represent the preferred choice, but treatment and restoration of normal pigmentation can be challenging [1]. Hydroquinone is considered the gold standard in pharmaceutical PIH treatment [3]; however, concerns with respect to the systemic absorption of hydroquinone and hydroquinone‐induced carcinogenesis have been expressed [8, 9] and hydroquinone also can cause exogenous ochronosis [10], a severe side effect which is very difficult to treat. Out of these safety issues, the European Union prohibited hydroquinone as a cosmetic ingredient in 2001 [11]. In the United States, the Food and Drug Administration is re‐evaluating the current status that formulations containing up to 2% hydroquinone are still sold as an over‐the‐counter drug [12]. A definitive decision is still pending. Therefore, safe and affordable cosmetic products for consumers with PIH are highly desirable. We recently identified isobutylamido‐thiazolyl‐resorcinol (Thiamidol) as an especially potent inhibitor of human tyrosinase [13, 14, 15]. To investigate if Thiamidol is effective and safe in the treatment of PIH, three studies were carried out. The first study examined the effect of Thiamidol on PIH in an epidermal wound model (suction blister‐induced PIH), while the second study analysed acne‐related PIH during a 12‐week vehicle‐controlled treatment with Thiamidol. Furthermore, a 12‐week clinical observational study was conducted in subjects with acne‐related PIH who applied a skin care regimen consisting of three different Thiamidol‐containing skin care products.

## MATERIAL AND METHODS

### Test formulations

For study I, isobutylamido‐thiazolyl‐resorcinol (Thiamidol) was formulated in an alcoholic gel without SPF. The formulation used in study II was an oil‐in‐water emulsion with Thiamidol and without SPF. For study III, volunteers utilized a skin care regimen consisting of three Thiamidol‐containing products (day cream SPF 30, night cream and dual serum).

### In vivo studies with Thiamidol‐containing formulations

#### Study I: Treatment of PIH induced by epidermal wounding (suction blister) with Thiamidol

A controlled, randomized, single‐centre study was conducted during summer in Germany (June to September) enrolling 14 healthy subjects with healthy, undamaged skin on the test sites (7 female, 7 male, aged 28 to 58 years) with Fitzpatrick skin phototypes II and III [16]. Exclusion criteria comprised chronic or acute skin diseases at the skin test sites, antibacterial, immunosuppressive, physical or cosmetic treatments of the skin test sites, intake of antihistamines or immunosuppressive medication, 14 days prior to the beginning of the study. Hormonal medication was not part of the exclusion criteria. The study was approved by a local ethic committee (approval no.: SP 4STU750, 011/1973), and the recommendations of the current version of the Declaration of Helsinki and the guideline of the International Conference on Harmonization Good Clinical Practice (ICH GCP) were observed, as applicable to a non‐drug study. All volunteers provided written, informed consent and all completed the study.

During the 2‐day preconditioning period, eligible subjects were required to stop using skin care products, as well as special skin care cleansing products such as shower oils, on the arms (inside and outside). Also, volunteers were asked to refrain from usage of tanning devices. Measurements were performed by trained and experienced personnel after acclimatization (21.5 ± 1.0°C and 45 ± 5% relative humidity).

On the first day of the study, two test areas were marked on one upper arm of the volunteers. Then, 2 suction blisters (9 mm diameter) were generated in each test area (4 × 2 cm) as previously described [17, 18]. Two weeks after blistering (i.e. one week after wound closure), baseline measurements (*T*_0_) were conducted. The skin colour of the suction blister sites and the surrounding skin was determined by Spectro‐pen^®^ (Dr. Bruno Lange GmbH, Düsseldorf, Germany). Additionally, digital images were taken using the Canon EOS‐1 equipped with a Canon Compact Macro Lens EF 50 mm and with a Canon Macro Ring Lite MR‐14EX (Canon, Tokyo, Japan). Remission spectrometry was performed using a Zeiss spectrometer MCS501 with a Xenon arc lamp. Original spectra of suction blister areas were normalized to spectra of surrounding normal skin. The positioning of vehicle and verum treatment was randomized and, subsequently, the investigator demonstrated correct application of the test formulas and trained the subjects in self‐application.

Subjects were asked to apply the respective test formulations twice daily over the next 12 weeks to the suction blister sites. The study was conducted blind with respect to the applied test formulations. Subjects were required to refrain from using skin care products on the arms during the entire study period. After 2, 5, 8 and 12 weeks, the skin colour of the suction blister PIH and the surrounding skin was determined by Spectro‐pen^®^ and photographs of the test areas were taken.

#### Study II: Treatment of post‐acne hyperpigmentation in Fitzpatrick phototype V subjects with Thiamidol

This single‐blinded, comparative, single‐centre clinical study was performed from June to October 2018 (darker period of the year from May to July) at Allergisa Pesquisa Dermato‐Cosmética LTDA, Campinas SP, Brazil. The study was conducted in conformance with the Declaration of Helsinki principles, the applicable regulatory requirements, including Resolution CNS no. 466/12, and in spirit of the Good Clinical Practices (Document of the Americas and ICH E6: Good Clinical Practice). Each subject provided written, informed consent and signed a photo release consent form authorizing the reproduction and distribution of any images collected during the study.

A total of 77 female subjects (aged 18 to 40 years), Fitzpatrick skin phototype V with a history of acne with remaining hyperpigmentation in the formerly affected area with no inflammatory acne lesion in the test area but at least 4 hyperpigmented spots (on whole face), met the inclusion criteria and were enrolled in the study, 64 participants completed the study. Exclusion criteria comprised topical acne therapy, skin diseases vitiligo, psoriasis and atopic dermatitis, intake of corticosteroids, immunosuppressive and anti‐histaminic drugs. Hormonal medication was not part of the exclusion criteria.

During the study, subjects were not allowed to apply skin‐lightening products to the face. They were further asked not to change any cosmetic or personal hygiene daily routine habits (including sunscreen, if used). Also, volunteers were asked to refrain from intense UV exposition (sunbathing, tanning devices).

At study start (*T*_0_), subjects were assessed by a dermatologist, who also performed a clinical grading. The subjects additionally answered a self‐grading questionnaire to evaluate their skin before the treatment. Also, one frontal and two lateral digital images of the face were taken using a VISIA CR photo station (Canfield Imaging Systems, Fairfield, NJ with a Canon Mark II 5D digital SLR camera (Canon, Tokyo, Japan).

Subjects were instructed to apply the respective test formulations twice daily for 12 weeks over the entire face (with the exception of inflammatory lesions) according to the provided use directions. The test formulas were randomly distributed to the subjects, 39 subjects applied the verum and 38 subjects the vehicle.

After 28 ± 2, 56 ± 2 and 84 ± 2 days of product application, subjects returned to the institute for evaluations. A dermatologist performed a control check of the acne status and a dermatological assessment of tolerance regarding possible sensations of discomfort. At the same points in time, subjects assessed their facial skin using the self‐grading questionnaire (based on the ‘Standard Guide for Sensory Claim Substantiation’ [19]). Subjects were requested to rate the visibility of hyperpigmentation compared to the surrounding facial skin by means of a scale ranging from 1 (clearly darker than surrounding, clearly defined – very well visible) to 10 (comparable colour than surrounding, overlaps into its surrounding – barely visible). Additionally, 3 facial images per subject were taken.

#### Study III: Treatment of PIH related to acne in Fitzpatrick phototype V and VI subjects with Thiamidol

This cosmetic clinical observational study was carried out at the dermatologist office of Prof. Ncoza Dlova, Durban, South Africa from September to December 2018 (brighter period of the year from November to February). Ethics approval was obtained from the Pharma‐Ethics Independent Research Ethics Committee, South Africa (Ref: 180419938). Subjects were screened for inclusion and exclusion criteria, and informed consent forms were signed by eligible participants. Volunteers also signed a photo release consent form authorizing the reproduction and distribution of any images collected during the study. 32 subjects (4 male and 28 female, aged 18 to 50 years) with acne‐related post‐inflammatory hyperpigmentation were enrolled in the study. Out of these, 29 completed the study. All volunteers were self‐reported as of African ancestry and were classified as Fitzpatrick skin phototypes V (24 subjects) or VI (5 subjects). Exclusion criteria comprised acne medication, aesthetic procedures 6 months prior to enrolment, usage of depigmenting products 2 months before beginning of the study or topical medication for the hyperpigmentation 30 days prior to the study entry. Hormonal medication was not part of the exclusion criteria. The subjects applied each of the three regimen products at home once daily (dual serum and day cream SPF 30 in the morning, night cream in the evening) for 12 weeks to the whole face according to usual skin care application habits. Participants were instructed to refrain from usage of any other creams for the duration of the study, except face cleanser and sunscreen, if used.

At baseline and again after 4, 8 and 12 weeks, the dermatologist determined the subjects’ skin profile and performed a clinical grading of efficacy and objective irritation parameters. Also, dermatologist questionnaires and patient self‐assessments were completed at baseline and after 4, 8 and 12 weeks. The investigator assessed skin evenness by means of the following 5‐point score: extreme (score 0), severe (score 1), moderate (score 2), slight (score 3) and absent (score 4). Also, the investigator evaluated the question: ‘How do you rate the improvement of the overall skin condition of the patient?’ using the 4‐tiered rating scale (very good, good, moderate, none).

Subjects determined their facial skin condition by means of a scale ranging from 1 (poor) to 10 (excellent). In addition, clinical photography (Canon EOS 1Ds Mark III, objective lens EF 50 mm 2.5 Makro, Canon Inc. Tokyo, Japan) and skin colour measurements of lesions and perilesional areas (Mexameter MX 18, Courage and Khazaka, Köln, Germany) were conducted at every visit.

### Statistical analysis

#### Study I

All statistical tests were 2‐sided at significance level alpha = 0.05. Statistical evaluation was carried out by means of the paired t‐test. Statistical analyses were performed using the Microsoft Excel build‐in functions.

#### Study II

Comparisons between time points and initial time point were performed through Wilcoxon Signed rank test. The bilateral hypothesis was used.

Comparisons between treatments were performed through Mann–Whitney test. The bilateral hypothesis was used. For each time points, comparisons between the two treatments were performed via Mann–Whitney test using differences to baseline (*T*_0_). The bilateral hypothesis was utilized.

For binary data, the two‐sided binomial test for null hypothesis proportion *p* = 0.5 was performed to test if the relative frequency for one category differs significantly from 0.5. Statistical analyses were performed using the XLSTAT 2018 software.

#### Study III

The Wilcoxon's signed rank test was used for the comparison of visits (two‐sided hypothesis testing, significance level 0.05, adjustment for multiple testing with the Bonferroni–Holm method). The analysis of the data was performed using Stata/IC 14.2 for Windows.

## RESULTS

**FIGURE 1:**
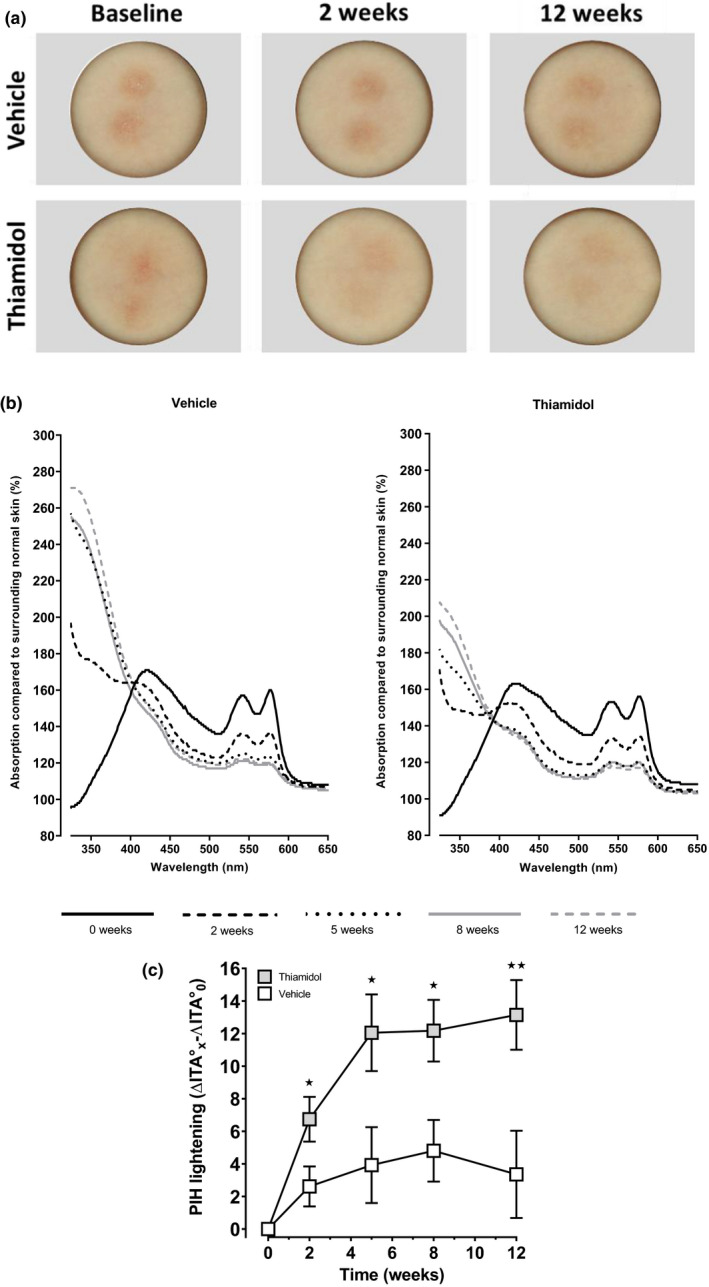
Treatment of experimentally induced suction blister PIH. (a) Representative examples of suction blister‐induced PIH treated with the Thiamidol‐containing formulation or vehicle at baseline and after 2 and 12 weeks of treatment. (b) Absorption spectra of suction blister‐induced PIH in comparison to surrounding normal skin. Spectra were taken at baseline and after 2, 5, 8 and 12 weeks of treatment. (c) ΔITA° values of suction blister‐induced PIH after treatment with the Thiamidol‐containing formula or vehicle at baseline and after 2, 5, 8 and 12 weeks of treatment. Data are depicted as mean ± SEM. Significant differences are marked in comparison to vehicle (⋆*p* < 0.05, ⋆⋆*p* < 0.01)

Suction blistering was performed to induce standardized areas of post‐inflammatory hyperpigmentation (PIH). Figure 1a presents examples of clinical pictures showing the development over time. At baseline (2 weeks after blistering, 1 week after wound closure), the skin colour of the test sites was still dominated by redness. At later points in time, redness decreased, and melanin dominated the skin colour, clearly visible at the vehicle‐treated site, barely at the Thiamidol‐treated site. Reflection spectra revealed a constant decrease of absorption by haemoglobin (~520–600 nm) and an increase of absorption by melanin, especially in the UV range. The latter was much more pronounced at the vehicle‐treated sites (Figure 1b). ITA° values were calculated from colour measurements, since this best reflects skin pigmentation and is not influenced by skin redness. After two weeks, the difference between vehicle and verum was already statistically significant (Figure 1c). On average, Thiamidol‐treated sites were lighter after 2 weeks than vehicle‐treated sites after 12 weeks. Suction blisters treated with the Thiamidol‐containing formulation showed statistically significant improvement compared with vehicle at all points in time (week 2: *p* = 0.034, week 5: *p* = 0.023, week 8: *p* = 0.011, week 12: *p* = 0.009).

**FIGURE 2:**
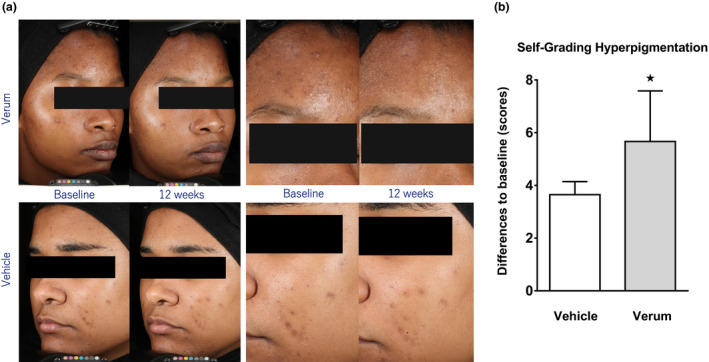
Treatment of acne‐induced PIH in dark‐skinned individuals. (a) Representative images of subjects with acne‐induced PIH at baseline and after 4, 8 and 12 weeks of treatment with the Thiamidol‐containing formulation (verum) or the vehicle. (b) Visibility of hyperpigmentation as determined by self‐grading after 4, 8 and 12 weeks of treatment with the Thiamidol‐containing formulation or the vehicle. Data are depicted as mean ± SD. Significant differences are marked in comparison to vehicle (⋆*p* < 0.05)

The relevance of the results of the experimental suction blister PIH model was further investigated in two studies with treatment of post‐acne marks. The photographs in Figure 2a show the improvement of acne‐related PIH after 12 weeks of treatment with a Thiamidol‐containing formulation. In this vehicle‐controlled, single‐blinded, single‐centre study, 39 subjects with phototype V applied the Thiamidol‐containing formulation and 38 the vehicle formulation. The cosmetic treatment was well tolerated, and no itching, burning, redness or other adverse effects were reported.

A statistical comparison of the two treatments revealed that the Thiamidol treatment produced a significantly (*p* = 0.047) stronger improvement for the visibility of hyperpigmentation after 12 weeks (compared with the vehicle treatment (Figure 2b).

**FIGURE 3:**
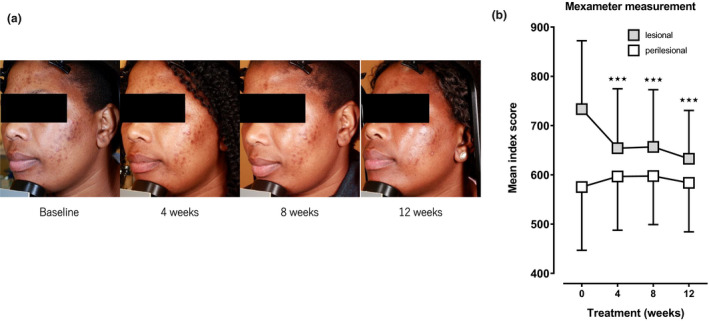
Treatment with a skin care regimen of three Thiamidol‐containing products. (a) Representative images of a subject at baseline and after 4, 8 and 12 weeks of treatment with the Thiamidol‐containing skin care regimen (day cream, night cream and dual serum). (b) Melanin index scores of lesional and perilesional skin after 4, 8 and 12 weeks of treatment. Data are depicted as mean ± SD. Significant differences are marked in comparison to baseline (⋆⋆⋆*p* < 0.001)

The efficacy of a Thiamidol‐containing skin care regimen on acne‐induced PIH was tested in a clinical observational study, also on subjects with phototype V‐VI. The effect of the Thiamidol‐containing formulation or vehicle on acne‐induced PIH after 4, 8 and 12 weeks of usage is shown in Figure 3a. Compared to baseline values (733.4 ± 138.8), the melanin index score of lesions dropped significantly (654.1 ± 120.6) after 4 weeks of treatment. After 8 weeks, a score of 656.7 ± 116.1 was documented, and at the end of the treatment period, a score of 632.7 ± 97.9 was observed. Compared with baseline, improvements were statistically significant for all points in time (*p* < 0.001). The perilesional area showed baseline values of 575.3 ± 128.6. The score of the perilesional area displayed no significant changes during the treatment period (4 weeks: 596.6 ± 109.1, 8 weeks: 597.5 ± 98.6, 12 weeks: 583.7 ± 99.5, Figure 3b).

**FIGURE 4:**
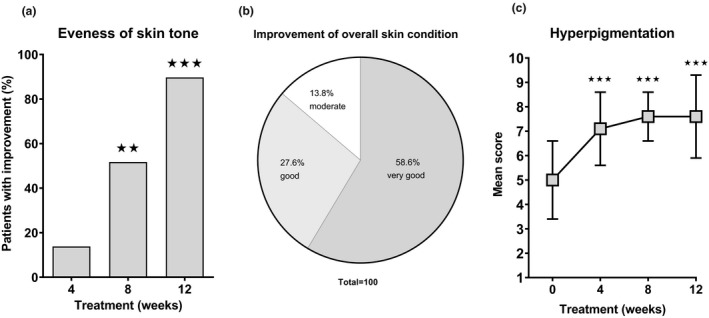
Expert rating and self‐assessment of facial skin condition. (a) Skin evenness as determined by the investigator at baseline and after 4, 8 and 12 weeks of treatment with the Thiamidol‐containing skin care regimen (day cream, night cream, dual serum). Data are depicted as mean ± SD. Significant differences are marked in comparison to baseline (⋆⋆*p* < 0.01, ⋆⋆⋆*p* < 0.001). (b) Overall skin condition rated by the investigator after 12 weeks of treatment with the Thiamidol‐containing skin care regimen. (c) Subjective assessment of hyperpigmentation at baseline and after 4, 8 and 12 weeks of treatment with the Thiamidol‐containing skin care regimen. Significant differences are marked in comparison to baseline (⋆⋆⋆*p* < 0.001)

Skin evenness was assessed by the investigator showing a continuous increase in the percentage of patients with improvement (13.8% after 4 weeks, 51.7% after 8 weeks and 89,7% after 12 weeks) (Figure 4a). As Figure 4b depicts, the improvement of the overall skin condition after 12 weeks of treatment was rated by the dermatologist as ‘very good’ in 58.6%, as ‘good’ in 27.6% and as ‘moderate’ in 13.8% of subjects.

Subjects conducted a self‐grading of their facial skin condition with respect to hyperpigmentation at baseline with a mean value of 5.0 ± 1.6. This value increased to 7.1 ± 1.5 after 4 weeks to 7.6 ± 1.0 after 8 weeks and to 7.6 ± 1.7 at the end of the study (Figure 4c). Compared to baseline, values were statistically significant for all study points in time (*p* < 0.001). Additionally, subjects assessed their acne‐induced PIH agreeing or disagreeing to the statement ‘My dark spots appear less pronounced’. The agreement rate increased from visit to visit and was 100% at the end of the study (86.2% after 4 weeks, 89.7% after 8 weeks and 100% after 12 weeks). Results from tolerability evaluations showed that all Thiamidol‐containing formulations were all well tolerated, the investigator determined the overall tolerability as excellent.

## DISCUSSION

PIH is an extremely common skin condition, can develop at any age and is not gender specific. Although it is usually temporary, it can take months or even years for the skin to return to a normal pigmentation. In some cases, the pigment irregularities can even be permanent [1].

Hyperpigmentation can be diminished by different processes [20], but the inhibition of melanin production, by blocking the key enzyme tyrosinase, is considered being the most effective and safest way. In the past, mushroom tyrosinase [21, 22] was used for testing potential tyrosinase inhibitors. However, this approach yielded inhibitors with unsatisfactory clinical results in humans, probably because mushroom tyrosinase differs significantly from human tyrosinase [23]. Using a recombinant human tyrosinase [24], recently the thiazolyl resorcinol Thiamidol was identified as a particularly potent and selective inhibitor [13, 14]. *In vitro,* Thiamidol was demonstrated to be superior to well‐known melanogenesis inhibitors such as arbutin, kojic acid and hydroquinone [13]. Clinically, treatment with Thiamidol at concentrations as low as 0.1% diminished the appearance of age spots. In women suffering from facial hyperpigmentation, 0.2% Thiamidol significantly improved dyspigmentation after 4 weeks of application [15].

Based on these data, we carried out two clinical and one clinical observational study to determine if Thiamidol is also effective and safe in the treatment of PIH.

First, we investigated the efficacy in experimentally induced suction blister PIH during 12 weeks of treatment with Thiamidol. Already after 2 weeks of application, suction blister sites treated with Thiamidol were significantly lighter than control sites and improved throughout the treatment period. At all points in time, suction blisters treated with the Thiamidol‐containing formula showed significantly lower skin colour differences between the suction blister PIH and the surrounding skin than the ones treated with vehicle. For practical reasons, this study was conducted in subjects belonging to the Fitzpatrick phototypes II and III, the predominant phototype in Hamburg, Germany where this study was performed. As shown in this suction blister study, PIH can occur in lighter skin; however, it is more common in darker pigmented skin (Fitzpatrick phototypes IV to VI). In individuals with darker pigmented skin phototypes, PIH is commonly observed in resolving acne lesions. The occurrence of PIH in individuals with acne was found to be 65% in African‐Americans, 53% in Hispanics and 47% in Asians [1]. A study including 324 subjects suffering from acne in seven Asian countries demonstrated that pigmentation problems often lasted at least 1 year in more than half of the subjects and 5 years or longer in 22% [7]. Individuals regard acne‐related pigmentation sometimes as even more burdensome than acne lesions [3], especially, since PIH can worsen with repeated inflammation [25].

In a second study, we therefore investigated the efficacy of a Thiamidol treatment in acne‐related PIH in darker‐skinned volunteers (Fitzpatrick phototype V). As determined by self‐grading, the Thiamidol treatment reduced the visibility of hyperpigmentation after 12 weeks to a significantly greater extent than vehicle treatment.

The treatment of PIH in patients with Fitzpatrick skin phototypes IV to VI represents a dermatological challenge, constitutes a long‐term treatment and always poses the risk that treatment‐associated irritation may aggravate PIH [1]; thus, it is essential that the formulation is well tolerated, which is the hallmark of cosmetic products.

For the previous studies, we used prototype formulations containing Thiamidol. Based on these formulations, a skin care regimen was developed, and tolerability and efficacy of three products (day care, night care and dual serum) were investigated in another study. Since UV exposure is an aggravating element in PIH [6], the regimen’s day cream contained a SPF 30 to minimize the potential for worsening hyperpigmentation due to sun exposure. The practical relevance of this regimen was analysed in an observational study in a dermatology office in Durban, South Africa, in subjects with Fitzpatrick phototypes V to VI exhibiting acne‐related PIH.

Compared with baseline values, the melanin index score of Thiamidol‐treated lesions showed significant improvements after 12 weeks of treatment with the new regimen. Interestingly, the surrounding skin did not show any lightening. Since PIH can cause emotional and psychological distress, the subjective assessment of efficacy is crucial. The subjects’ self‐assessment confirmed an excellent rating regarding the improvement of hyperpigmentation.

In summary, Thiamidol significantly improved not only suction blister‐induced PIH, but also acne‐related pigment irregularities in darker‐skinned individuals. Thiamidol was excellently tolerated and very well accepted. Thus, Thiamidol is an effective and safe new cosmetic agent to treat PIH.

## CONFLICT OF INTEREST

Dennis Roggenkamp, Tobias Mann, Jan Batzer, Julia Riedel, Martina Kausch, Ivica Zoric and Ludger Kolbe are employees of Beiersdorf AG. Thiamidol is patented by Beiersdorf AG. None of the other authors state a conflict of interest.
